# The Emerging Roles of Protein Lipidation in Fertility and Reproductive Disorders: Mechanisms and Therapeutic Implications

**DOI:** 10.3390/biom16030389

**Published:** 2026-03-05

**Authors:** Heran Cao, Xiaohua Liu, Shenghui Zhu, Hua Nie, Shujuan Liu, Jun Fan, Weibing Qin

**Affiliations:** 1 Department of Medical Biochemistry and Molecular Biology, School of Medicine, Jinan University, Guangzhou 510632, China; caohr@gdszjk.org.cn; 2 The NHC Key Laboratory of Male Reproduction and Genetics, Guangdong Provincial Reproductive Science Institute, Guangzhou 510632, China; liuxh@gdszjk.org.cn (X.L.); zshenghui2026@126.com (S.Z.); nieh@gdszjk.org.cn (H.N.); 3 College of Animal Science and Technology, Northwest A&F University, Yangling 712100, China; 2025390069@gzhmu.edu.cn; 4 Key Laboratory of Viral Pathogenesis & Infection Prevention and Control, Jinan University, Ministry of Education, Guangzhou 510632, China

**Keywords:** protein lipidation, post-translational modifications, male reproduction, female reproduction, infertility, therapeutic targets

## Abstract

Protein lipidation, a pivotal post-translational modification, dynamically regulates cellular signaling, membrane trafficking, and protein stability, with emerging roles in reproductive physiology. However, the systematic understanding of how distinct lipid modifications orchestrate physiological processes in male and female reproduction remains incomplete. This review systematically elaborates the mechanistic contributions of protein lipidation to gametogenesis, fertilization, and reproductive homeostasis. Finally, we discuss emerging therapeutic strategies targeting lipidation pathways—including inhibitors of palmitoylation (2-BP) and prenylation (lonafarnib)—and highlight their potential for treating infertility and reproductive disorders.

## 1. Introduction

Protein lipidation refers to a group of post-translational modifications where lipid molecules are covalently attached to proteins, significantly enhancing their hydrophobicity and altering their cellular localization, stability, and interactions [1,2]. This modification is integral to a variety of cellular processes, including signal transduction, membrane targeting, and protein–protein interactions [3]. Protein lipidation plays a crucial regulatory role in many physiological processes, including reproductive biology, where it influences cellular signaling, hormone responses, and gametogenesis [4,5,6]. The five primary types of protein lipidation are palmitoylation [7], myristoylation, prenylation, glycosylphosphatidylinositol (GPI) anchoring, and cholesterylation [8]. Each of these modifications attaches distinct lipid groups to specific amino acid residues and regions, thereby influencing the protein’s function in distinct ways. Finally, cholesterylation is a modification in which cholesterol is covalently attached to specific proteins, particularly in pathways such as Hedgehog signaling, thereby regulating protein distribution and activity [9]. In both male and female reproductive systems, lipid modifications are known to regulate the localization and function of key proteins involved in fertility [10,11,12,13] (Table 1 and Table 2). For example, lipidated proteins regulate sperm functional integrity, ATP production in flagella, and folliculogenesis via membrane localization and signaling cascades. Despite growing recognition of lipidation’s role in cellular signaling, its contribution to reproductive disorders remains poorly understood, presenting both a challenge and an opportunity for targeted therapeutic development. In this study, a systematic literature search was conducted using the PubMed, Web of Science, and Google Scholar databases (period: 1982–2025). The search employed keyword combinations such as “S-palmitoylation, N-myristoylation, S-prenylation, O-octanoylated, or C-terminal GPI Anchoring AND sperm, testis, spermatogenesis, epididymis, sperm maturation, deferent duct, or fertilization” and “S-palmitoylation, N-myristoylation, S-prenylation, O-octanoylated, or C-terminal GPI Anchoring AND ovary, oocyte, uterus, oviduct, oogenesis, or oocyte maturation”, yielding 1302 relevant entries. Inclusion criteria were as follows: (1) studies directly investigating the role of lipidation modifications in the reproductive system; (2) articles addressing molecular mechanisms or clinical relevance; and (3) publications in peer-reviewed journals. After excluding redundant and duplicate entries, 109 articles were selected for in-depth analysis, focusing on groundbreaking discoveries and controversial perspectives from the past five years. This review explores the mechanisms by which lipidation affects reproductive functions, shedding light on the importance of these modifications in processes such as oocyte maturation, sperm function, and hormonal signaling. By providing a detailed overview of current research, we aim to identify potential targets for lipidation-based therapeutic interventions in reproductive disorders.

## 2. Protein Lipidation in Male Reproduction

### 2.1. S-Palmitoylation

**Figure 1:**
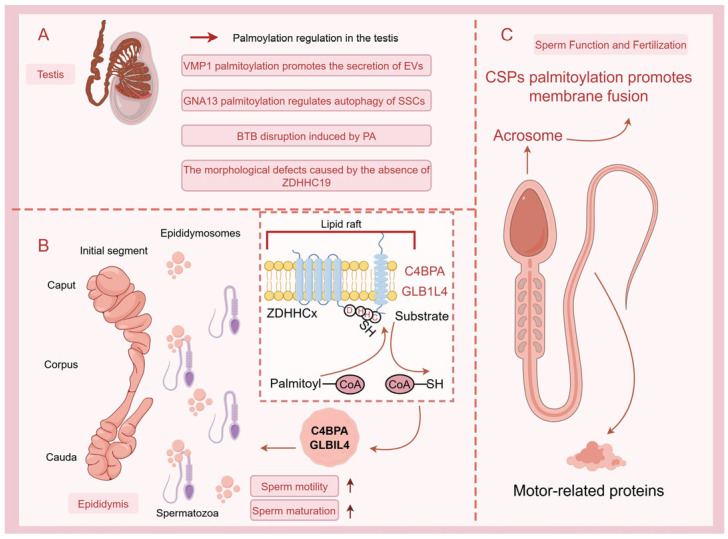
Schematic diagram illustrating the key roles of protein S-palmitoylation in the male reproductive system (created with Figdraw, www.figdraw.com). This figure systematically illustrates the core regulatory mechanisms of reversible protein S-palmitoylation modification during spermatogenesis, maturation, and functional acquisition. (**A**) Palmitoylation regulatory network in the testes. The dynamic palmitoylation modification is jointly regulated by palmitoyltransferases (PATs, such as the ZDHHC family) and depalmitoylases (APTs) in the testicular microenvironment. (**B**) Palmitoylation and sperm maturation in the epididymis. The epididymis, divided into the initial segment, head, body, and tail, is a critical site where sperm acquire motility and fertilization capacity. Epididymosomes enriched with palmitoylated proteins in the epididymal fluid serve as important carriers for protein transport. Proteins such as GLB1L4 and C4BPA, enriched in epididymosomes via palmitoylation modification, enhance sperm motility. (**C**) Palmitoylation in sperm and fertilization function. Mature sperm function relies on the palmitoylation modification of key proteins. The palmitoylation of cysteine-string proteins (CSPs) located in the sperm acrosomal region is essential for their anchoring to the membrane structure. This modification regulates acrosomal exocytosis during fertilization by promoting membrane fusion, playing a decisive role in sperm membrane stability and successful fertilization, and potentially serving as a clinical diagnostic biomarker.

S-palmitoylation, a dynamic and reversible post-translational modification in contrast to the generally irreversible nature of other lipidations, mediates the attachment of palmitate to cysteine residues via thioester bonds, dynamically regulating protein membrane localization and signaling cascades [41]. This process is catalyzed by DHHC-type palmitoyltransferases (PATs) and reversed by acyl-protein thioesterases (APTs), forming a regulatory circuit critical for cellular homeostasis [42]. In male reproduction, such lipid modifications govern essential processes, including spermatogenesis, sperm-egg fusion, and motility by modulating key reproductive proteins’ stability and trafficking. In the testis, multiple palmitoylated proteins have been identified to play critical roles in spermatogenesis. Proteomic characterization of palmitoylated proteins in mouse testes has revealed that a substantial number of these proteins are functionally associated with sperm motility, spermatogenesis, and sperm movement [43]. Treatment with palmitic acid (PA) in mice was found to induce blood-testis barrier (BTB) disruption. Further studies revealed that PA-induced dysregulation of protein palmitoylation plays a pivotal role in BTB impairment and subsequent spermatogenic dysfunction. Clinically, elevated serum PA levels were significantly observed in patients with non-obstructive azoospermia (NOA) and extreme oligospermia (EO) [6]. Another clinical study demonstrated the critical link between defective protein palmitoylation and impaired testicular organogenesis, where a homozygous G287V mutation in HHAT (encoding Hedgehog acyltransferase) disrupted Desert Hedgehog (DHH)/Sonic Hedgehog (SHH) palmitoylation, resulting in 46, XY Disorders of Sex Development (DSD) with testicular dysgenesis and skeletal defects. This provides direct evidence for the essential role of Hedgehog lipid modification in human gonadal development [44]. The importance of palmitoylation in regulating signal transduction is particularly evident in mediating the communication between Sertoli cells (SCs) and spermatogonial stem cells (SSCs) within the testis. Research has demonstrated that palmitoylation of VMP1 in SCs enhances the secretion of extracellular vesicles (EVs) and effectively promotes the growth of SSCs [45]. Moreover, our recent study has revealed that DHHC13 mediates S-palmitoylation of GNA13 at Cys14 and Cys18 residues in SCs, promoting its enrichment in SC-derived EVs. Notably, palmitoylated GNA13 in EVs significantly downregulates autophagy levels in SSCs [16]. These findings demonstrate that palmitoylation participates in SCs-SSCs communication via exosomal pathways and contributes to testicular microenvironmental homeostasis. In SCs, conditional knockout of palmitoyl-protein thioesterase 1 (PPT1)—a depalmitoylase—leads to impaired sperm quality and increased malformation rates. Specifically, PPT1 deficiency causes abnormal lysosomal accumulation and elevated cholesterol levels in SCs, along with reduced adhesion between germ cells and SCs, ultimately resulting in decreased male fertility [17]. In addition to the aforementioned palmitoylated proteins, the PATs also play a critical role in regulating spermatogenesis. Studies have demonstrated that ZDHHC19 plays a crucial role in maintaining sperm function in mice, although it appears to be dispensable for spermatogenesis per se. Knockout studies reveal that Zdhhc19-deficient mice exhibit normal spermatogenesis but develop significant sperm abnormalities, including defective head and tail morphology, reduced motility, and impaired acrosome reaction, ultimately resulting in male infertility [14,15]. These findings indicate that ZDHHC19-mediated palmitoylation is essential for the post-spermatogenesis stages, where it contributes to the functional integrity of sperm. These studies suggest that palmitoylated proteins may serve as potential therapeutic targets for male infertility. Beyond its implications for infertility treatment, palmitoylation—as a reversible post-translational modification—may also hold promising applications in the development and implementation of non-hormonal male contraceptives. Recent studies have demonstrated that the TEX38-ZDHHC19 heterodimer regulates sperm head morphogenesis via ZDHHC19’s PAT activity, providing a druggable target for non-hormonal contraceptive development [46]. Additionally, palmitoylation has been implicated in the epigenetic inheritance of traits through sperm. Specifically, the partitioning of paternal epigenetic inheritance (PEI) granules to spermatids, which is essential for paternal epigenetic inheritance, is dependent on palmitoylation, highlighting the broader implications of this modification beyond sperm development to intergenerational transmission of epigenetic information [19]. The epididymis contains numerous proteins with specific expression patterns, which are critical for sperm maturation as they are transferred to the sperm surface [47]. We have conducted some studies on palmitoylated proteins in the epididymis. In rats, we found that palmitoylated beta-galactosidase-like protein (GLB1L4) is transported via exosomes from the caput to the cauda of the epididymis. The palmitoylation status of GLB1L4 affects its cellular localization and interaction with sperm, suggesting that this modification is vital for the proper maturation and functionality of sperm as they transit through the epididymis [18]. Furthermore, our recent findings in mice demonstrate that the complement component 4 binding protein (C4BPA), expressed in the caput epididymis, is enriched in epididymosomes via palmitoylation modification, thereby enhancing sperm motility [10]. These findings provide a mechanistic basis for the role of palmitoylated proteins in epididymal sperm maturation. Although clinical evidence regarding epididymal palmitoylation remains lacking, our results may support the development of therapeutic strategies for epididymis-related male infertility as well as post-meiotic male contraceptives targeting the epididymis. Finally, palmitoylation influences the function of cysteine string proteins (CSPs) in human sperm, which are involved in membrane fusion processes critical for acrosomal exocytosis during fertilization. CSPs, especially the beta isoform enriched in the testis, rely on palmitoylation for membrane attachment, and this modification is essential for their role in promoting sperm membrane stability and facilitating the fusion events necessary for successful fertilization and may also serve as a biomarker for the clinical diagnosis of infertility [48]. Overall, the evidence strongly suggests that palmitoylation is a key regulatory modification in male reproductive physiology, impacting various stages from spermatogenesis to sperm function and fertility. Further studies on palmitoylation could uncover novel therapeutic targets for treating male infertility (Figure 1).

### 2.2. N-Myristoylation

N-myristoylation, a major protein lipidation type, involves covalent attachment of myristate (C14 fatty acid) to N-terminal glycine via amide bonds, with rare lysine modifications reported. This predominantly co-translational modification can also occur posttranslationally during apoptosis, critically influencing protein stability, membrane targeting, and protein–protein interactions [49]. Although studies on N-myristoylation in male reproduction remain limited, this modification—like palmitoylation—plays crucial roles in regulating membrane signaling. One notable example is the sperm-specific hexokinase 1 isoform (HK1S), which undergoes N-myristoylation at its N-terminus, a modification critical for its atypical localization to the plasma membrane [20]. This membrane association is essential for the compartmentalized glycolytic activity required to generate ATP locally, thereby supporting sperm motility. The myristoylation of HK1S, coupled with palmitoylation, facilitates its membrane tethering, ensuring efficient energy production in the flagellum, which is crucial for sperm function [20]. These findings suggest that sperm function may be co-regulated by multiple lipid modifications, implying that clinical interventions targeting protein lipidation might require combinatorial approaches for optimal therapeutic efficacy. Similarly, a novel dual specificity protein phosphatase (DSP) known as VHY, which is highly expressed in the testis, also undergoes N-myristoylation. This modification is vital for its localization to the plasma membrane, where VHY likely plays a role in the regulation of signaling pathways during spermatogenesis [21]. Moreover, the flagellar creatine kinase (TCK) in sea urchin sperm, which is involved in the phosphocreatine shuttle—a system essential for energy transport and sperm motility—is another protein that relies on N-myristoylation for its membrane association. Studies have demonstrated that TCK exists in both myristoylated and non-myristoylated forms, with the myristoylated variant showing a stronger association with lipid membranes, thereby ensuring its localization to the flagellum where it facilitates energy transfer critical for sperm motility [22,23]. Current evidence indicates that N-myristoylation plays essential roles in membrane signal transduction of specific sperm proteins, functionally analogous to palmitoylation. Although clinical data directly linking N-myristoylation to male reproductive outcomes remain unavailable, future therapeutic applications may parallel those developed for palmitoylation modulation.

### 2.3. Acylation of Other Saturated Fatty Acids (O-Octanoylated)

O-octanoylation, a lipid modification primarily observed in ghrelin, which requires O-octanoylation at serine-3 for its endocrine actions, plays a role in various physiological processes, including growth hormone release and appetite regulation. The enzyme responsible for this modification is called ghrelin O-acyltransferase (GOAT) [50]. GOAT serves as the exclusive enzyme catalyzing ghrelin acylation, a prerequisite for the bioactive form to engage growth hormone secretagogue receptor 1a (GHS-R1a) [51]. Ghrelin is widely expressed in the male testis, epididymis, prostate, and seminal vesicle [52]. Moreover, GHSR-1a protein is detected in the Golgi apparatus, acrosome, and acrosomal region of rat spermatozoa, as well as on the cell membrane of epididymal sperm heads [53]. These findings suggest a crucial regulatory role of Ghrelin signaling in male reproduction. Functionally, studies have demonstrated that Ghrelin, through its antioxidant capacity, effectively alleviates ischemia/reperfusion [54] or doxorubicin [55]-induced testicular injury in rats and mitigates oxidative stress caused by varicocele, thereby shortening the spermatogenic cycle [56]. In the context of testicular torsion, a condition known to cause significant testicular damage, the expression of octanoylated ghrelin and NUCB2/nesfatin-1 was investigated. The study found that testicular torsion led to a significant increase in the expression of both octanoylated ghrelin and NUCB2/nesfatin-1, reflecting the extent of tissue damage in rats [24]. Notably, treatment with N-acetylcysteine (NAS) mitigated the torsion-induced increase in octanoylated ghrelin levels, especially in the early stages of torsion, highlighting the potential therapeutic role of octanoylated ghrelin in preventing torsion-related infertility [24]. In the epididymis, Ghrelin counteracts the detrimental effects of a fructose-enriched diet on sperm quality in rats by upregulating the expression of glutathione peroxidase 3 (Gpx3) [25]. In mice, both the expression of GHS-R1α and its endogenous ligand ghrelin are upregulated in the testes of Leptin-deficient (ob/ob) animals. Pharmacological blockade of the ghrelin signaling pathway restored androgen synthesis, mitigated germ cell apoptosis, and ultimately enhanced sperm output in ob/ob mice [57]. Additionally, ghrelin exerted partial protective effects against cisplatin [58] or cyclophosphamide [59]-induced testicular injury in male mice in a GHS-R1α-dependent manner. These findings collectively suggest functional conservation of ghrelin’s reproductive regulatory roles across species. Clinically, patients with idiopathic non-obstructive azoospermia (INOA) exhibit significantly elevated serum ghrelin levels compared to fertile controls [60]. Notably, ghrelin is quantifiable in human seminal plasma, though its concentrations are markedly diminished compared to serum levels. Importantly, seminal plasma ghrelin levels do not differ significantly between normospermic and dyspermic men. Although Ghrelin signaling appears pivotal for male reproductive function, clinical studies remain scarce regarding the specific role of O-octanoylated Ghrelin in spermatozoa [61]. Future clinical investigations may explore the therapeutic potential of exogenous Ghrelin administration as an antioxidant to enhance semen quality or as a cryoprotective agent for sperm preservation.

### 2.4. S-Prenylation

S-prenylation, a critical post-translational modification, mediates membrane targeting of proteins via attachment of farnesyl (15C) or geranylgeranyl (20C) isoprenoids to C-terminal cysteine residues through thioether bonds. This modification predominantly regulates small GTPases, with farnesylation governing H-/N-Ras localization and signaling, while geranylgeranylation directs Rho/Rab membrane association via lipid-binding partners. Beyond GTPases, S-prenylation is essential for heterotrimeric G proteins and nuclear lamins, with dysregulation implicated in aberrant signaling and disease pathogenesis [62]. Alterations in the balance of protein prenylation have been shown to significantly impact spermatogonial differentiation and stem cell maintenance. For instance, the deletion of geranylgeranyl diphosphate synthase (Ggpps) in germ cells or Sertoli cells leads to infertility due to abnormal spermatogonial differentiation and the depletion of SSCs during the prepubertal stage. This depletion is primarily driven by enhanced farnesylation of Rheb, which activates the mTORC1 pathway, accelerating differentiation and inducing apoptosis in spermatogonia [13]. Additionally, disruptions in protein prenylation within Sertoli cells, such as those induced by Ggpps deficiency, have been linked to inflammatory responses that lead to long-term infertility. The altered farnesylation of H-Ras in these cells results in excessive cytokine and chemokine production, stimulating spermatogonial apoptosis and macrophage invasion into the seminiferous tubules, further exacerbating germ cell loss [26]. These findings highlight the importance of maintaining a precise balance between protein geranylgeranylation and farnesylation in the seminiferous epithelium, particularly during the early stages of spermatogenesis. The disruption of this balance can lead to structural and functional impairments in the BTB and germ cell adhesion, which are critical for normal spermatogenesis and male fertility [27].

### 2.5. C-Terminal GPI Anchoring

Glycosylphosphatidylinositol (GPI) anchoring represents a widespread eukaryotic posttranslational modification that tethers proteins to the outer leaflet of the plasma membrane. GPI-anchored proteins (GPI-APs) critically regulate diverse biological processes [63]. As transcriptionally inactive cells, sperm may acquire GPI-APs through surface “coating” to mediate cell–cell communication processes. To date, twenty-nine GPI-APs have been identified in human and mouse testes, among which GLIPR1L1, SPAM1, and SPACA4 have been functionally characterized to mediate sperm-egg interactions [64,65,66]. Male mice deficient in either TEX101 or LY6K exhibit identical infertility phenotypes, although these sterile males can produce offspring through in vitro fertilization (IVF) [67,68]. However, the precise molecular mechanisms underlying the infertility of these sperm remain elusive. Additionally, while other GPI-anchored proteins such as CD55 and CD59 have been identified, gene-targeted knockout mice lacking these proteins exhibit normal viability and fertility [69,70]. A recent study revealed that the sperm adhesion protein IZUMO1, while predominantly characterized for its essential role in sperm-egg fusion, demonstrates intrinsic fusogenic activity that does not require interaction with its canonical GPI-anchored receptor JUNO [28,29].

In the epididymis, spermatozoa must acquire GPI-Aps to ensure their maturation and subsequent fertilization competence. The transfer of GPI-Aps to the sperm surface occurs via two distinct pathways: a clusterin (CLU)-dependent soluble, membrane-free mechanism and a CD9-mediated exosome-based pathway [71]. Including several well-characterized epididymal luminal proteins: SPAM1 [72] (critical for acrosome reaction initiation), GLIPR1L1 [73] (involved in zona pellucida binding), and additional members of the hyaluronidase family, such as HYAL2 [74], HYAL3 [75], and HYAL5 [76]. However, the modification mechanisms of these GPI-APs on epididymal sperm and their transfer processes within the epididymal lumen require further investigation. Intriguingly, studies have identified that sperm acquire several immune-related marker proteins (e.g., CD52, CD55, CD59, CD73) during epididymal transit, which may protect them from immune attacks in both male and female reproductive tracts [30]. This protective role was experimentally confirmed in a study demonstrating that goat sperm GPI-APs shield against macrophage-mediated phagocytosis [77], although the precise molecular mechanisms remain elusive. During sperm capacitation, the release of CD52 has been demonstrated to induce substantial membrane reorganization, which is essential for triggering the acrosome reaction and subsequent fertilization [78,79]. Clinical studies have demonstrated that CD52 participates in both clot formation and liquefaction processes in human semen [80]. Furthermore, CD52 levels demonstrate a significant negative correlation with sperm motility [81]. The CD52 isoform SAGA-1 has been identified on sperm surfaces of infertile patients and may contribute to immunological infertility pathogenesis. Notably, the glycosylation differences between SAGA-1 and CD52 could represent potential pathogenic factors for immunological infertility and other autoimmune disorders [82,83]. To summarize the key lipidated proteins and their roles discussed in this section, please refer to Table 3.

## 3. Protein Lipidation in Female Reproduction

### 3.1. S-Palmitoylation

S-palmitoylation plays an essential regulatory role in female reproductive physiology, influencing various cellular processes within ovarian follicular cells and impacting fertility. Several studies have highlighted the involvement of S-palmitoylation in folliculogenesis, oocyte maturation, and steroidogenesis. For example, palmitoylation in granulosa cells (GCs) is critical for regulating protein stability, sorting, and signaling, thereby affecting oocyte competence and maturation. Specifically, palmitoylation of proteins such as G-proteins and estrogen receptors within the follicle promotes key signaling pathways that support oocyte development [11]. Moreover, enzymes such as ZDHHC17 and PPT1 play pivotal roles in maintaining the palmitoylation status of heat shock proteins, such as HSP90α, which are crucial for androgen-to-estrogen conversion. Dysregulation of this process has been linked to reproductive disorders such as polycystic ovary syndrome (PCOS), where reduced palmitoylation impairs estrogen synthesis and exacerbates hyperandrogenism [31]. Additionally, research has shown that palmitoylation facilitates membrane binding of critical proteins, such as Ha-Ras, thereby enhancing their ability to regulate meiotic maturation in oocytes [32]. In addition to its role in steroidogenesis and oocyte maturation, palmitoylation also influences granulosa cell viability. Palmitic acid, when excessively accumulated, can induce granulosa cell apoptosis through lipotoxic pathways, ultimately leading to follicular atresia and reduced egg production [33]. This highlights the delicate balance required in lipid metabolism for maintaining ovarian function. In clinical studies, APT1 and APT2 expression levels have been found to be significantly elevated in the uterine tissues of women with adenomyosis. This upregulation subsequently increases the S-palmitoylation of Scribble, a polarity protein, thereby promoting its translocation from the basolateral membrane to the cytoplasm [84]. In the future, certain reproductive disorders arising from dysregulated expression of palmitoylating enzymes may be amenable to enzyme replacement therapy (ERT). This approach involves the intravenous administration of recombinant palmitoylating enzymes produced in vitro, thereby restoring physiological S-palmitoylation homeostasis in affected tissues.

### 3.2. S-Prenylation

Protein prenylation plays a critical role in regulating female reproductive processes, including ovarian function, steroidogenesis, and granulosa cell survival. In the context of PCOS, insulin resistance and oxidative stress are key factors contributing to hyperandrogenism, with the mevalonate pathway playing a pivotal role. The isoprenylation of small GTPases like Ras and Rac, which are critical for cell proliferation and signaling, is disrupted in hyperinsulinemia, leading to the enhanced steroidogenesis observed in PCOS [34]. Statins, by inhibiting the mevalonate pathway, reduce the prenylation of these proteins, thereby diminishing oxidative stress and the proliferative effects of insulin and IGF-I, offering therapeutic potential for managing PCOS by addressing both hyperinsulinemia and hyperandrogenism. Consequently, the clinical administration of statins effectively ameliorates dyslipidemia associated with PCOS. In vitro, depletion of substrates essential for protein prenylation—specifically farnesyl pyrophosphate (FPP) and geranylgeranyl pyrophosphate (GGPP)—in granulosa cells has been demonstrated to induce apoptosis, thereby underscoring the critical role of prenylation in maintaining cell viability during ovulation and luteinization [12]. Moreover, the farnesylation of InsP3 5-phosphatase promotes membrane interactions, altering its activity and subsequently modulating calcium oscillations in granulosa cells [35]. Studies on human ovarian granulosa cells have demonstrated that depletion of substrates for protein prenylation elevates cellular apoptosis levels [12]. Investigations in mice have revealed that Ggps1 deficiency disrupts the geranylgeranylation pathway—a process critical for small GTPase activation—ultimately leading to impaired uterine contractions and dystocia [36]. Furthermore, the isoprenylation of proteins, such as inositol 1,4,5-trisphosphate (InsP3) 5-phosphatase, regulates calcium signaling, which is vital for numerous cellular processes, including folliculogenesis and ovulation [37].

### 3.3. C-Terminal GPI Anchoring

**Figure 2:**
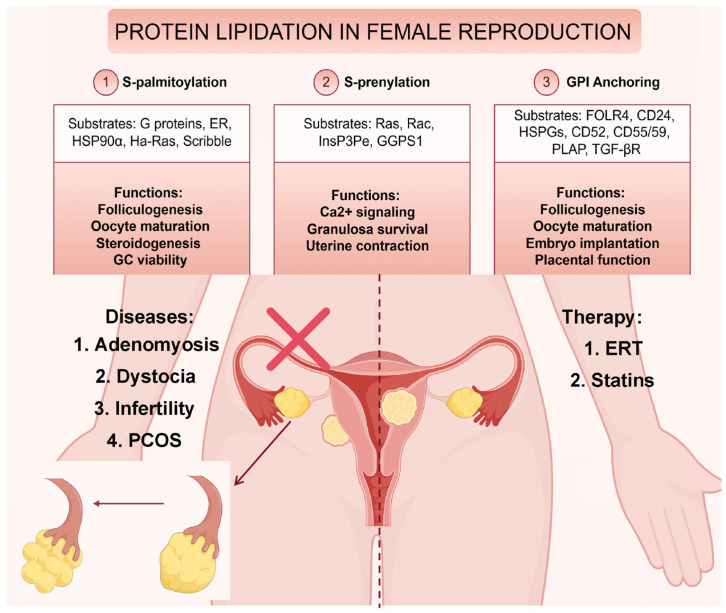
Schematic diagram illustrating the key roles of protein lipidation in the female reproductive system (created with Figdraw, www.figdraw.com). This figure summarizes the molecular mechanisms and clinical significance of three major types of protein lipidation—S-palmitoylation, S-prenylation, and GPI anchoring—within the female reproductive system. The upper section of the figure presents three modules detailing the substrate proteins and specific ovarian functions for each lipidation type: S-palmitoylation primarily regulates folliculogenesis, oocyte maturation, and steroidogenesis; S-prenylation is involved in calcium signaling, granulosa cell survival, and uterine contraction; GPI-anchored proteins play crucial roles in follicular development, embryo implantation, and placental function. The central part features a schematic anatomical diagram of the female reproductive system, with red “X” marks indicating target organs associated with pathology. An inset in the lower left corner contrasts the morphology of a normal ovary versus a polycystic ovary. The figure also summarizes four major diseases linked to dysregulated protein lipidation (adenomyosis, dystocia, infertility, and polycystic ovary syndrome) and proposes corresponding therapeutic strategies, including enzyme replacement therapy (ERT) and statin intervention.

GPI-APs are equally pivotal in female reproductive physiology, orchestrating processes from folliculogenesis to embryo implantation. In the ovary, GPI-APs such as folate receptor 4 (FOLR4) and CD24 are enriched on oocyte membranes, where they mediate bidirectional communication between oocytes and granulosa cells. FOLR4, a GPI-anchored folate transporter, is essential for oocyte maturation and early embryonic development by regulating folate metabolism, a deficiency of which leads to infertility in female mice [38]. CD24, another GPI-AP, is critically involved in ovulatory triggering in GCs. Notably, CD24^+^ cumulus GCs exhibit a significantly reduced proportion in PCOS patients compared to controls. These findings collectively suggest that CD24 may serve as a potential therapeutic target for addressing ovulatory dysfunction [39]. Clinically, CD24 has emerged as a prognostic and diagnostic biomarker for ovarian cancer [85]. In addition, other GPI-APs, including heparan sulfate proteoglycans (HSPGs) [86], HYAL1 [87], HYAL3 [87], and CLU [88], play critical roles in modulating follicular atresia—a process essential for ovarian follicle quality control. At the maternal-fetal interface, GPI-anchored proteins like CD52 have been implicated in uterine receptivity during embryo implantation, with the regulation of CD52 being linked to transcription factors like NKX2.2 [89]. Moreover, the GPI-anchored transforming growth factor-beta (TGF-β) receptors on human endometrial cells mediate critical signaling pathways, impacting endometrial cell proliferation and differentiation during the menstrual cycle [90]. CD55 [91] and CD59 [92], expressed on endometrial epithelial cells, inhibit complement activation to prevent immunological rejection of the semi-allogeneic embryo. Clinically, hCG administration modulates the expression levels of CD55 and CD59 in the endometrial microenvironment, thereby regulating embryo-endometrial crosstalk critical for embryo implantation. Furthermore, placental alkaline phosphatase (PLAP), a GPI-anchored hydrolase, modulates angiogenesis and nutrient transport in the placenta [40]. A summary of the pivotal lipid-modified proteins and pathways involved in female reproductive processes is provided in Table 4 and Figure 2.

## 4. Therapeutic Applications of Small-Molecule Modulators of Protein Lipidation in Male and Female Reproduction

### 4.1. Palmitoylation Inhibitor

Small-molecule modulators of protein lipidation have emerged as promising therapeutic tools for addressing reproductive dysfunctions in both males and females. In male reproduction, inhibitors targeting S-palmitoylation, such as 2-Bromopalmitate (2-BP), have led to dysfunction of the male mouse reproductive system and inflammatory responses, accompanied by changes in the gut microbiota [93]. In addition, the significant decrease in sperm motility in mice treated with 2-BP suggests that inhibiting palmitoylation may negatively affect male reproductive capacity [10]. For females, 2-BP inhibits the stimulatory effect of luteinizing hormone (LH) on progesterone synthesis in isolated luteal cells, suggesting that fatty acid oxidation partially mediates the steroidogenic effect of LH in ovarian steroidogenesis [94]. The mechanism of 2-BP action involves multiple pathways affecting membrane protein function and lipid metabolism. Primarily, 2-BP may indirectly modulate the catalytic activity of integral membrane proteins, such as PATs, by altering the lipid microenvironment. Its intracellular conversion to 2-BP-CoA yields a non-hydrolyzable analog that competitively inhibits enzymatic activity by forming a stable inhibitor-enzyme complex, thereby blocking substrate transfer to acceptor proteins. Furthermore, 2-BP depletes intracellular palmitoyl-CoA pools—the essential substrate for protein palmitoylation—thereby disrupting post-translational protein modification processes [95].

Current investigations into protein O-palmitoylation have predominantly centered on Wnt signaling molecules, which require site-specific lipid modification at hairpin 2 mediated by porcupine (PORCN), an endoplasmic reticulum (ER)-localized membrane-bound O-acyltransferase. This enzymatic process, essential for functional maturation of Wnt proteins, involves the covalent attachment of a palmitoleoyl group to confer signaling competency [96]. PORCN, a member of the membrane-bound O-acyltransferase (MBOAT) family, catalyzes acyl group transfer from either acyl-CoA donors or accessory proteins to lipid/protein substrates [97]. LGK-974 is an o-palmitoylation inhibitor that reduces FSH-stimulated estradiol production in bovine granulosa cells by inhibiting both canonical and noncanonical WNT pathways, but the effects of o-palmitoylation on female reproduction have not been reported [98]. The LGK-974 occupies a cytosolic hydrophobic pocket stabilized by residues Phe246, Val297, Trp300, Thr328, Tyr329, Leu349, and Leu408, with Ser332 forming a hydrogen bond to its carbonyl oxygen. This binding mode is conserved across potent PORCN inhibitors, which typically feature analogous L-shaped conformations and oxygen-containing pharmacophores to mimic the 4′-phosphopantetheine-fatty acid moiety of palmitoleoyl-CoA. Mechanistically, structural overlap between LGK974 and palmitoleoyl-CoA’s acyl-donor domain suggests competitive inhibition through active site occupation, thereby blocking catalytic transfer of lipid groups [99]. While LGK-974 remains unexplored for reproductive applications clinically, this PORCN inhibitor is currently under clinical investigation for cancer treatment [100,101], having demonstrated potent anti-proliferative effects in colorectal cancer cell lines in vitro [102]. In Phase I clinical trials, LGK-974 exhibited a manageable safety profile characterized by dose-dependent dysgeusia (50% overall incidence) and bone metabolism abnormalities (6% incidence), which are considered on-target effects resulting from systemic WNT pathway inhibition [101]. Although the reproductive consequences of aberrant O-palmitoylation remain undefined, clinical reports have identified homozygous missense variants in MBOAT1, another O-acyltransferase, associated with non-obstructive azoospermia. Notably, no documented cases link PORCN dysfunction to fertility disorders in humans or model organisms [103]. This knowledge gap suggests potential therapeutic utility of LGK-974 in addressing PORCN-related reproductive pathologies pending further mechanistic validation (Figure 2).

### 4.2. Prenylation Inhibitor

Lonafarnib, a farnesyltransferase inhibitor, competitively blocks the CAAX motif of target proteins to suppress prenylation, thereby preventing the farnesylation-dependent accumulation of pathogenic progerin and progerin-like proteins in Hutchinson-Gilford Progeria Syndrome (HGPS) and related laminopathies [104]. Although lonafarnib has not been clinically investigated for reproductive applications, it received U.S. Food and Drug Administration (FDA) approval in 2020 for reducing mortality risk in HGPS and treating processing-deficient progeroid laminopathies associated with heterozygous LMNA mutations or homozygous/compound heterozygous ZMPSTE24 mutations [105]. Clinically, lonafarnib exhibits a manageable safety profile, with common adverse events including vomiting (90%), diarrhea (81%), and infections (78%), though severe risks include hepatotoxicity (elevated ALT/AST in 27–35% of patients) and hypertension [6]. Preclinical studies suggest potential regulatory effects of lonafarnib on female reproductive pathways in animal models. Studies have demonstrated that the combination of Lonafarnib and paclitaxel effectively inhibits the proliferation of murine ovarian cancer [106]. Given the critical regulatory role of prenylation in PCOS, Lonafarnib may represent a potential adjuvant therapeutic candidate for PCOS management. However, further rigorous investigation in animal and cellular models is required to validate its efficacy and mechanistic underpinnings.

**Figure 3:**
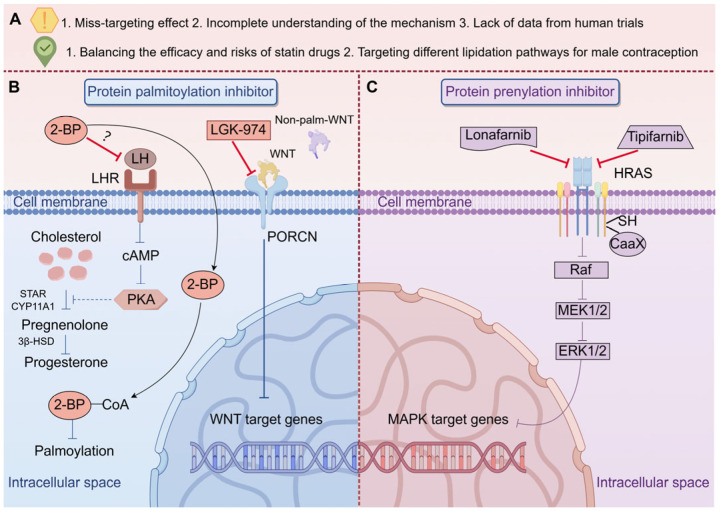
Molecular mechanisms and therapeutic prospects of small-molecule modulators targeting protein lipidation in reproductive processes. This figure illustrates the mechanisms of action of two main classes of small-molecule lipidation modulators, and outlines the potential challenges and future directions for their application in reproductive medicine (created with Figdraw, www.figdraw.com). (**A**) Current challenges and future therapeutic directions. (**B**) Protein palmitoylation inhibitors. The left panel illustrates the mechanism of action of S-palmitoylation inhibitors. 2-Bromopalmitate (2-BP), as a broad-spectrum inhibitor, is converted intracellularly into 2-BP-CoA, a non-hydrolyzable analog that competitively inhibits palmitoyl acyltransferases (PATs) and depletes the palmitoyl-CoA pool. This disrupts the palmitoylation process of associated proteins; 2-BP may also interfere with LHR and downstream cAMP/PKA signaling. LGK-974 is a specific inhibitor of Porcupine (PORCN), the enzyme responsible for O-palmitoylation of Wnt proteins. By blocking the maturation and secretion of Wnt ligands, LGK-974 can inhibit the Wnt signaling pathway, thereby suppressing target gene expression. (**C**) Protein prenylation inhibitors. The right panel illustrates the mechanism of action of farnesyltransferase inhibitors (FTIs). Both lonafarnib and tipifarnib competitively inhibit farnesyltransferase, preventing CAAX motif-containing proteins (such as HRAS) from binding to farnesyl isoprenoid lipids. This inhibition blocks the membrane localization and subsequent activation of HRAS, thereby disrupting the downstream Raf-MEK1/2-ERK1/2 MAPK signaling cascade and transcription of MAPK target genes.

Tipifarnib, an orally administered non-peptidomimetic farnesyltransferase inhibitor, has demonstrated clinical efficacy in diverse malignancies. The agent recently received FDA approval based on Phase II/III trial results demonstrating its therapeutic potential in elderly patients with acute myeloid leukemia (AML) [107]. Mechanistically, tipifarnib exerts its inhibitory effect by blocking the post-translational modification process mediated by farnesyltransferase, which facilitates the membrane localization of signaling proteins. Notably, while all RAS isoforms contain farnesyltransferase binding sites, HRAS exhibits unique metabolic dependency—its membrane localization function is exclusively dependent on farnesylation. This selective mechanism enables tipifarnib to specifically displace membrane-associated HRAS, thereby disrupting MAPK signaling cascade activation [108]. Studies demonstrate that tipifarnib induces apoptotic cell death in human periovulatory ovarian granulosa cells, implicating its regulatory role in germ cell survival through disruption of protein prenylation pathways [12]. HRAS plays a pivotal role in regulating pre-ovulatory granulosa cell function [109]. Epidemiological evidence demonstrates that childhood mumps virus infection is significantly associated with elevated H-Ras farnesylation in GGPPS-deficient Sertoli cells, which mechanistically drives constitutive activation of MAPK and NF-κB signaling pathways, ultimately leading to acquired infertility associated with germ cell developmental defects [26]. Given the pathological mechanisms underlying HRAS signaling dysregulation in reproductive system disorders, oral tipifarnib therapy targeting farnesyltransferase inhibition may represent a novel adjuvant treatment approach. Therapeutic modulation of protein lipidation in reproduction is a nascent yet promising frontier. However, their clinical utility remains limited due to off-target effects, incomplete mechanistic understanding, and lack of human trials. For instance, while 2-BP disrupts sperm motility and steroidogenesis in mice, its broader toxicity complicates therapeutic use. Similarly, statins reduce hyperandrogenism in PCOS but may impair granulosa cell survival. Despite these challenges, the ability to selectively target lipidated pathways—such as geranylgeranylation in spermatogonial differentiation or GPI-anchored proteins in embryo implantation—offers a unique opportunity to develop precision therapies (Figure 3).

## 5. Conclusions and Future Perspectives

Protein lipidation plays a pivotal role in reproductive physiology, regulating processes from gametogenesis to fertilization through dynamic post-translational modifications such as S-palmitoylation, N-myristoylation, S-prenylation, and GPI anchoring. While these modifications are essential for sperm function, oocyte competence, and hormonal signaling, their dysregulation contributes to infertility and reproductive pathologies. It is important to acknowledge that current lipidomics and high-throughput (HT) proteomic analyses of protein lipidation are still evolving technologies. This knowledge gap stems largely from the limitations of prevailing analytical methodologies. Widely used HT approaches, such as the ABE for S-acylation, can identify modification sites but typically do not reveal the specific identity of the bound lipid. Moreover, these methods can be prone to false-positive identifications. This is particularly evident in the context of sperm biology, where our understanding remains largely skewed towards S-acylation (e.g., S-palmitoylation), primarily due to the availability of robust enrichment methods like ABE for thioester-linked modifications. In contrast, the analysis of other lipid modifications (e.g., N-myristoylation, O-octanoylation) at a proteome-wide scale is severely hampered by the lack of equally specific and efficient enrichment tools. Therefore, future progress in HT analytical technologies—such as the development of novel chemoproteomic probes and enrichment strategies for diverse lipid moieties—and parallel advances in analytical chemistry that couple precise site identification with direct lipid characterization, are pivotal. These advancements will be key to uncovering a more complete and definitive landscape of sperm protein lipidation, potentially revealing novel, non-S-palmitoylation modifications that are critical for fertility and yet to be discovered.

Key unresolved questions include the crosstalk between lipidated proteins and other post-translational modifications, the tissue-specific roles of lipidation enzymes, and the potential of lipidomics for early disease diagnosis. With advancing research on lipidation modifications in reproductive biology, future studies should explore a broader spectrum of small-molecule inhibitors, facilitating their clinical translation as adjuvant therapies. The synergistic application of these inhibitors in combination with alternative therapeutic strategies will significantly expand the therapeutic armamentarium in reproductive medicine. RNA interference-mediated therapeutic approaches hold promise for achieving tissue-specific attenuation of pathogenic lipidated proteins in pathological conditions such as PCOS. Complementarily, metabolic intervention strategies targeting upstream lipid donor pathways, particularly mevalonate pathway inhibition, may enable systemic modulation of protein prenylation while minimizing off-target effects through enhanced pharmacological specificity. Lipidomics combined with single-cell transcriptomics could map spatiotemporal lipidation patterns during spermatogenesis. Epigenetic-lipidomic crosstalk analysis may reveal novel regulatory nodes in ovarian aging. These multi-modal therapeutic strategies should be prioritized for clinical translation. Collaborative efforts integrating reproductive biology with nanotechnology and artificial intelligence-driven drug design will accelerate the development of precision reproductive medicine.

## Figures and Tables

**Table 1:** Regulation of Male Reproduction by Lipid Modifications.

Lipidation Type	Role in Male Fertility	Unanswered Questions
S-Palmitoylation	• Sperm motility (ZDHHC19 [14,15], GNA13 [16], PPT1 [17]) • BTB integrity [6] • Epididymal sperm maturation (GLB1L4 [18], C4BPA [10]) • Paternal epigenetic inheritance [19]	• ZDHHC family functional diversity • Acyl-protein thioesterase specificity • Non-spermatogenic roles in the testis
N-Myristoylation	• HK1S: flagellar ATP production [20] • VHY: phosphatase localization during spermatogenesis [21] • TCK: energy transport during sperm motility [22,23]	• Dual lipidation crosstalk • Clinical relevance in infertility
O-Octanoylation	• testicular damage [24] • Sperm antioxidation [25]	• O-octanoylated Ghrelin in spermatozoa • Ghrelin isoform functions
S-Prenylation	• SSCs maintenance (Rheb/mTORC1 [13]) • Inflammatory regulation (H-Ras [26]) • BTB structural integrity [27]	• Prenyltransferase inhibitor selectivity • Metabolic compensation mechanisms
C-terminal GPI Anchoring	• Sperm-egg fusion (IZUMO1 [28,29]) • Epididymal immune protection (CD52 [30])	• GPI-AP shedding regulation • Immune evasion mechanisms

**Table 2:** Regulation of Female Reproduction by Lipid Modifications.

Lipidation Type	Role in Female Fertility	Unanswered Questions
S-Palmitoylation	• PCOS (HSP90α [31]) • Oocyte meiosis (Ha-Ras [32]) • Granulosa cell apoptosis [33]	• PCOS therapeutic window • Follicular atresia pathways
S-Prenylation	• PCOS [34] • Granulosa cell apoptosis [35] • Uterine contraction (Ggps1 [36]) • Folliculogenesis and ovulation (InsP3 [37])	• Ovulation timing regulation • Endometrial receptivity links
C-terminal GPI Anchoring	• Oocyte maturation (FOLR4 [38]) • PCOS(CD24 [39]) • Placental nutrient transport (PLAP [40])	• Glycan shielding mechanisms • Maternal-fetal interface crosstalk

**Table 3:** Key Lipidated Proteins and Their Roles in Male Reproduction.

Protein	Type of Lipidation	Expression/Localization	Role in Male Reproduction
GNA13	S-palmitoylation	Sertoli cells	SCs-SSCs communication, testicular microenvironment homeostasis [16]
GLB1L4	S-palmitoylation	Epididymis	Epididymal sperm maturation [18]
C4BPA	S-palmitoylation	Caput epididymis	Enhancement of sperm motility [10]
VMP1	S-palmitoylation	Sertoli cells	SCs-SSCs communication, SSC growth promotion [45]
DHH/SHH	S-palmitoylation	Hedgehog signaling pathway	Testicular organogenesis and sex development [44]
CSPs	S-palmitoylation	sperm	Sperm membrane stability, acrosomal exocytosis during fertilization [48]
HK1S	N-myristoylation	Sperm plasma membrane/flagellum	Sperm motility [20]
VHY	N-myristoylation	Testis	Regulation of signaling pathways during spermatogenesis [21]
TCK	N-myristoylation	sperm flagellum	Energy transport and sperm motility [22,23]
Ghrelin	O-octanoylation	Testis, epididymis, spermatozoa	Alleviation of testicular injury, antioxidant effects [24,54,55]
H-Ras	S-farnesylation	Sertoli cells	Inflammatory response and acquired infertility [26]
GLIPR1L1/SPAM1/SPACA4	C-terminal GPI anchoring	Testis, sperm surface	Mediation of sperm-egg interactions [64,65,66]
TEX101/LY6K	C-terminal GPI anchoring	Testis, sperm	Mediation of sperm-egg interactions [67,68]
CD55/CD59/CD73	C-terminal GPI anchoring	Sperm	Potential immune protection [30]
GPI-APs	C-terminal GPI anchoring	Epididymal lumen	Sperm maturation and fertilization competence acquisition [71]
HYAL2/HYAL3/HYAL5	C-terminal GPI anchoring	Epididymal lumen	Acrosome reaction and zona binding [74,75,76]
CD52	C-terminal GPI anchoring	Sperm surface	Immune protection; Sperm capacitation and fertilization; Semen liquefaction; Potential clinical biomarker [30,77,78,79,80,81,82,83]

**Table 4:** Key Lipidated Proteins and Their Roles in Female Reproduction.

Protein	Type of Lipidation	Expression/Localization	Role in Male Reproduction
HSP90α	S-palmitoylation	Granulosa cells	Androgen-to-estrogen conversion, PCOS pathogenesis [31]
Ha-Ras	S-palmitoylation	Oocyte	Regulation of meiotic maturation in oocytes [32]
Scribble	S-palmitoylation	Endometrial/uterine tissues	Cell polarity, implicated in adenomyosis [84]
ER	S-palmitoylation	Follicular cells	Supporting oocyte development signaling [11]
Ras/Rac	S-prenylation	Granulosa cells	Cell proliferation, signaling, and steroidogenesis; implicated in PCOS pathogenesis [34]
InsP3	S-prenylation	Granulosa cells	Regulation of calcium signaling, affecting folliculogenesis and ovulation [35,37]
FOLR4	C-terminal GPI anchoring	Oocyte membranes	Oocyte maturation and early embryonic development [38]
CD24	C-terminal GPI anchoring	Oocyte membranes	Ovulatory triggering; Potential PCOS biomarker/therapeutic target; Ovarian cancer biomarker [39,85]
HSPGs/HYAL1 /HYAL3/CLU	C-terminal GPI anchoring	Ovary	Modulation of follicular atresia [86,87,88]
CD52	C-terminal GPI anchoring	Maternal-fetal interface	Uterine receptivity during embryo implantation [89]
TGF-β receptors	C-terminal GPI anchoring	Endometrial epithelial cells	Endometrial cell proliferation and differentiation during the menstrual cycle [90]
CD55/CD59	C-terminal GPI anchoring	Endometrial epithelial cells	Prevention of immunological rejection of the semi-allogeneic embryo [91,92]

## Data Availability

Data sharing is not applicable to this article as no new data were created or analyzed in this study. All information discussed is sourced from the publications cited in the reference list.
